# Practical Remediation of Hg-Contaminated Groundwater by MoS_2_: Batch and Column Tests

**DOI:** 10.3390/molecules29215132

**Published:** 2024-10-30

**Authors:** Haifeng Wang, Shuai Wei, Shuai Huang, Wei Liu, Zongwu Wang

**Affiliations:** 1 Kaifeng Key Laboratory of Food Composition and Quality Assessment, School of Environmental Engineering, Yellow River Conservancy Technical Institute, Kaifeng 475004, China; 2 Department of Science and Technology Evaluation Service, Henan Provincial Science Research Platform Service Center, Zhengzhou 450008, China

**Keywords:** remediation, mercury, groundwater, MoS_2_, adsorption

## Abstract

Trace mercury contamination in groundwater poses a serious threat to ecological systems and human health. The kinetics and isotherms of MoS_2_ (MS) for Hg removal were studied in batch tests under an unfavorable high salinity and low mercury environment. Flower-like MS with nanosheets can effectively remove Hg in the groundwater matrix, with a shorter equilibrium time (3 h), superior removal efficiency (94.26%), excellent distribution coefficient (5.69 × 10^6^ mL g^−1^), and higher maximum adsorption capacity (926.10 ± 165.25 mg g^−1^). Furthermore, the Adams-Bohart model (*R*^2^ = 0.9052–0.9416) can accurately describe the dynamic interception process of the initial stage (≤40 PVs), and the Yan model (*R*^2^ = 0.9765−0.9941) depicts the whole process (140 PVs) of MS in a fixed column well. A higher dosage of m, but lower *C*_0_ and *ν*_p_ facilitate the interception efficiency in column tests. Based on the characterizations of *X*-ray photoelectron spectroscopy (XPS) and scanning electron microscopy (SEM), which were used to simultaneously consider the species of Hg and the groundwater matrix, surface complexation, electrostatic attraction, ion exchange, and precipitation is a plausible interfacial adsorption mechanism of MS for mercury. The excellent performance demonstrates that MS with nanosheets is a promising candidate for the PRB remediation of trace Hg in saline groundwater.

## 1. Introduction

Groundwater and surface water are the most important reservoirs on Earth, and groundwater provides the majority of drinking and agricultural water [1]. However, groundwater contamination is increasing, posing a serious threat to human health and food safety [2]. Due to its lethal toxicity and non-biodegradability, the problem of heavy metal mercury pollution poses an even greater challenge. At the same time, the remediation of groundwater is to some extent hampered by high salinity and difficult accessibility [3]. Among the various technologies for groundwater remediation, permeable reactive barrier (PRB) technology stands out for its efficient in situ sustainable interception and low cost [4,5,6,7]. However, the reactive materials with high-performance, high stability, and low-cost have become the key to PRBs technology everywhere from the laboratory to the engineering project. Therefore, recently, many efforts have been made to develop more suitable materials, including zero valent iron (ZVI), activated carbons (AC), zeolites, lime and other alkaline materials, apatite, sodium dithionite, transformed red mud, oxides, materials for biobarriers, combinations of reactive materials, and others [8,9]. Unfortunately, regarding the removal of mercury from groundwater, the low availability and the poor performance remain unresolved.

An emerging two-dimensional nanomaterial, black powdered molybdenum disulfide (MS) with nanosheets exhibits abundant exposed sulfur atoms, strong covalent bonds (Mo−S) within the monolayer, and weak van der Waals forces between the monolayers. Based on the strong Lewis acid–base soft–soft interactions between Hg and S, the theoretical maximum adsorption capacity of MS can be up to 2506 mg L^−1^ [10,11]. Therefore, regarding research on the preparation of MS, the application and the mechanism of mercury removal have been deeply developed in recent years. The MS has a strong affinity for heavy metal ions (Hg^2+^ > Pb^2+^ > Cd^2+^ > Zn^2+^) and the strongest affinity for Hg under certain conditions [12]. The defective MS nanosheets decorated with Fe_3_O_4_ nanoparticles possess high selectivity and a superior capacity for Hg^2+^ removal, and the maximum adsorption capacity calculated by the Langmuir isotherm model is 425.5 mg g^−1^, which is higher than that of other reported magnetic thiol-modified adsorbents [13]. For the MS incorporated with 11% atomic oxygen, the adsorption capacity and rate can increase by more than 21 and 17 times, respectively. This is because the oxygen atom can facilitate the migration of the hydrated Hg^2+^ ions to MS surfaces and enhance the complexation between the S atom and Hg^2+^ [14]. Due to the stronger complexation between the S sites and the heavy metal, which involves oxidation-reduction, electrostatic attraction, and complexation between Hg^2+^ and MS, the synthesized multifunctional MS in a single-metal system showed a higher adsorption capacity for Hg^2+^ than that of Pb^2+^ and Cd^2+^ [15]. MS nanosheets can also capture Hg in groundwater with high selectivity, superior uptake capacity, an ultra-fast removal rate, and high stability. The substrate in which mercury is located could affect the performance and mechanism of mercury removal. For Hg in DI water, the strong affinity between Hg and MS via strong Lewis acid/base soft-soft interactions is the dominant mechanism. However, the dominant species of mercury for the presence of Cl^−^ in groundwater is HgClOH, and HgClOH can generate HgCl free radicals, which could reduce Hg^2+^ to Hg_2_Cl_2_ precipitate by dimerization or Hg^0^ by a chemical reduction reaction. All of this can increase the total removal capacity of Hg (up to 6288 mg g^−1^) by MS, which is higher than all previously reported values [16]. Furthermore, the flower-like MS can efficiently remove Hg^2+^ from the acidic wastewater, and the maximum uptake capacity was 1790 mg g^−1^ at 25 °C [17].

To improve the applicability of MS in engineering, various MS-based composite materials have become a hot topic. The hierarchical structure of MS and the surface coordination of Hg-S make the MS/Fe_3_O_4_ nanocomposite a promising Hg purifier with fast kinetics, high selectivity, a wide pH range, and superior uptake capacity (1923.5 mg g^−1^). In addition, the as-synthesized MS/Fe_3_O_4_ has excellent applicability, and the removal rate of Hg^2+^ (0–1000 μg L^−1^) in natural waters and industrial effluents can reach up to ~97% [18]. Rose-like MS can simultaneously remove the toxic metal ions (Hg, Pb, and Ag) from water, and the adsorption capacity for Hg (1991 mg·g^−1^) is significantly higher than those of Pb and Ag. The rose flower-shaped structure provides abundant active S^2−^ sites, and the strong affinity (between S^2−^ and metal ions) and the electrochemical factor facilitate the adsorption [19]. The recyclable and inexpensive polyvinylidene fluoride (PVDF) membranes decorated with MS-nanosheets can efficiently capture Hg^2+^ without being affected by ionic strength and coexisting anions, and the maximum adsorption capacity of 578 mg·g^−1^ can be achieved based on the main active sites of S [20]. To overcome the problems of aggregation and separation of powders, macroporous double-network MS-based beads (DMBs) were constructed via polymer crosslinking between calcium alginate and poly vinyl alcohol for the removal of Hg^2+^ from various matrices. The DMBs exhibited a high selectivity and capacity (253.6 mg g^−1^), which was attributed to the surface coordination of Hg−S and the formation of Hg(II) complexes [21].

In addition, inorganic MS with nanosheets can be synthesized by means of a simple hydrothermal–solvothermal reaction. However, there are no reports on the practical application of MS with the PRB technology for the remediation of Hg-contaminated groundwater, and the systematic study via batch and column experiments is almost nonexistent.

To verify the efficacy of flower-like MS in high salinity, low concentration Hg-contaminated groundwater and to assess the applicability of MS in practical PRB remediation for Hg removal, this study systematically investigated the removal performance, influencing factors, and breakthrough processes. The objectives of this work were: (1) to investigate the process of Hg sorption through kinetics and isotherms, qualitatively analyzing the matrix effects for Hg removal in groundwater (ionic strength, pH, and coexisting metal ions) via batch experiment; (2) to investigate the remediation performance of MS in PRBs for mercury interception in simulated groundwater through column tests, taking into account the effects of actual geological factors on groundwater; (3) to reveal plausible adsorption mechanisms, taking into account the experimental data for adsorption and characterization results of MS.

## 2. Results and Discussion

### 2.1. Characterizations of MS

**Figure 1:**
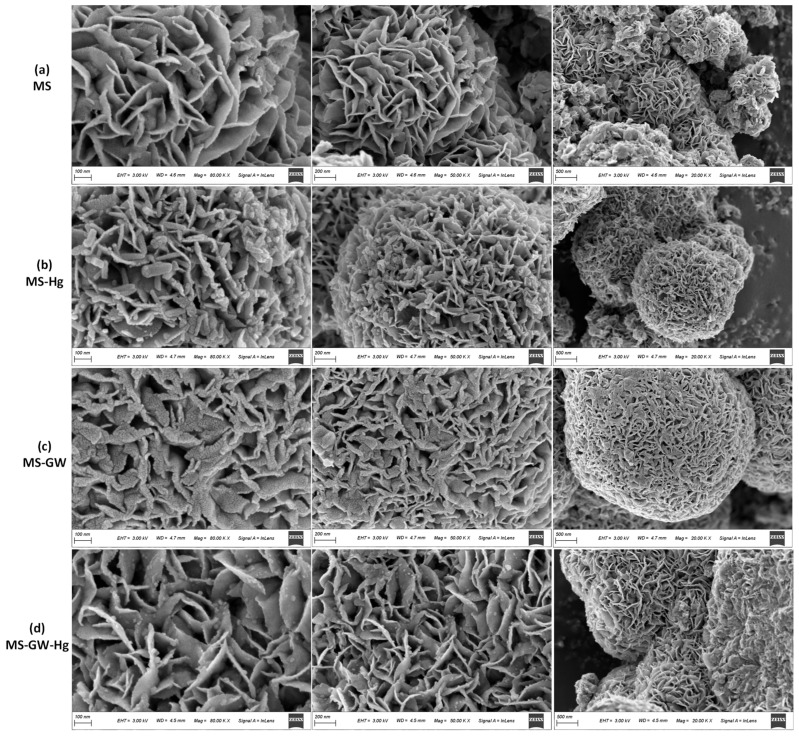
The SEM images of (**a**) MS, (**b**) MS-Hg (MS after adsorption in the DI water with mercury), (**c**) MS-GW (MS after adsorption in the simulated groundwater without mercury), and (**d**) MS-GW-Hg (MS after adsorption in the simulated groundwater with mercury).

**Figure 2:**
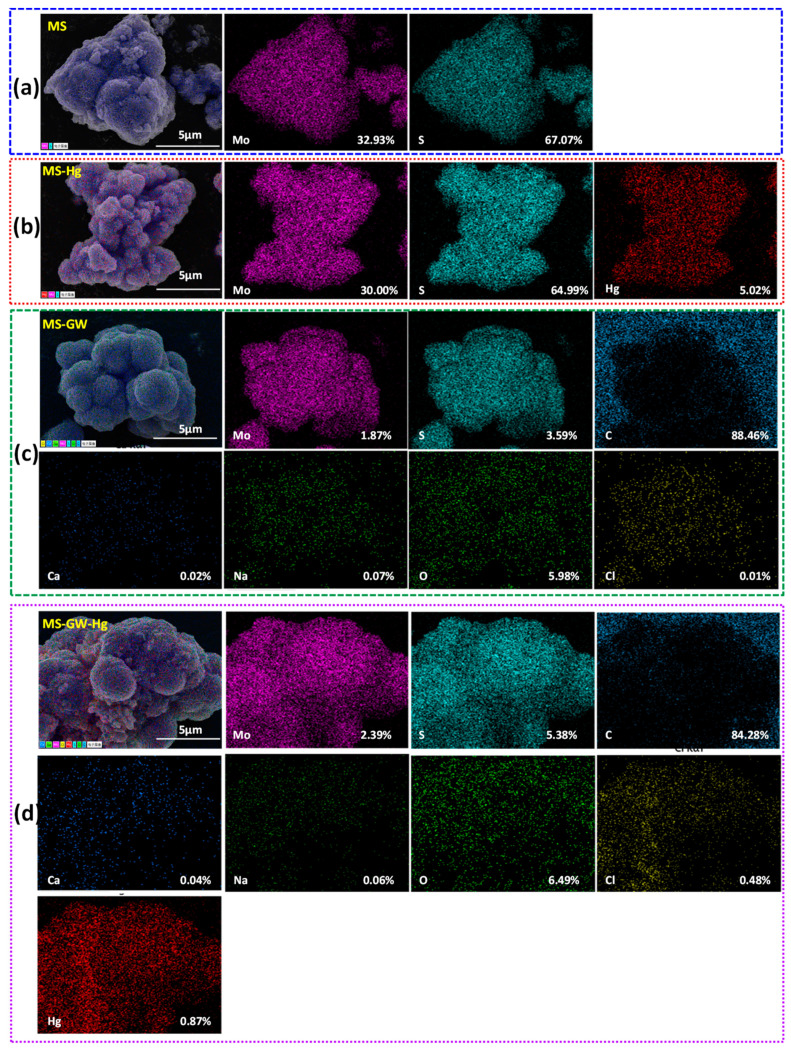
The SEM-mapping of (**a**) elements Mo and S for MS, (**b**) elements Mo, S, and Hg for MS-Hg, (**c**) elements Mo, S, C, Ca, Na, O, and Cl for MS-GW, and (**d**) elements Mo, S, C, Ca, Na, O, Cl, and Hg for MS-GW-Hg. The numbers in SEM-mapping represent atomic percentages.

**Figure 3:**
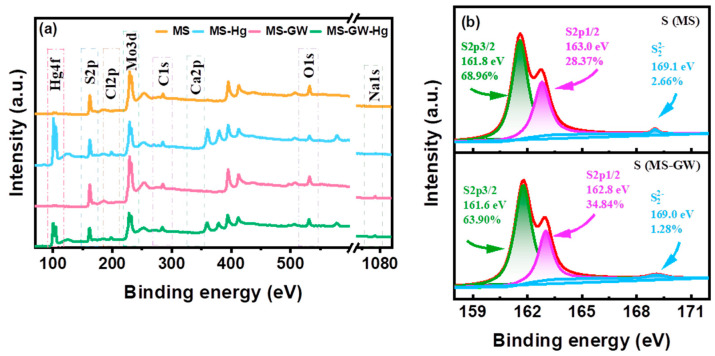
(**a**) The XPS survey spectra of MS, MS-Hg, MS-GW, and MS-GW-Hg; (**b**) high-resolution XPS spectrum of S2p for MS and MS-GW.

The as-synthesized MS exhibits a distinct spherical flower-like structure with a diameter of approximately 3–5 μm. The rose-shaped flower ball is composed of 10–20 nm thick nanosheets with interlayer gaps of 50–300 nm (Figure 1a). As shown in Figure S1, peaks at 2θ = 14.0°, 33.3°, 39.2°, 49.2°, and 59.3° for MS correspond well with the (002), (100), (103), (105), and (110) reflections (JCPDS No. 37-1492), which confirms the formation of hexagonal MS phase [22,23]. According to Figure 2a, Mo and S are evenly distributed on the surface of MS. As shown in Figure 3a, two peaks centered at 228.8 and 161.8 eV in the survey spectrum of MS represent the existence of Mo and S elements in MS, corresponding to Mo 3d and S2p, respectively. In the high resolution XPS spectrum of S2p (Figure 3b), there are three signals, which can be assigned to S2p3/2 of S^2−^ (161.8 eV, 68.96%), S2p1/2 of S^2−^ (163.0 eV, 28.37%), and $S_{2}^{2-}$ (169.1 eV, 2.66%) [11,22]. Considering the atomic ratio of Mo and S (Figure 2a), it can be inferred that molybdenum disulfide mainly exists in the form of MS.

### 2.2. Batch Hg Adsorption Processes in Simulated Groundwater

**Figure 4:**
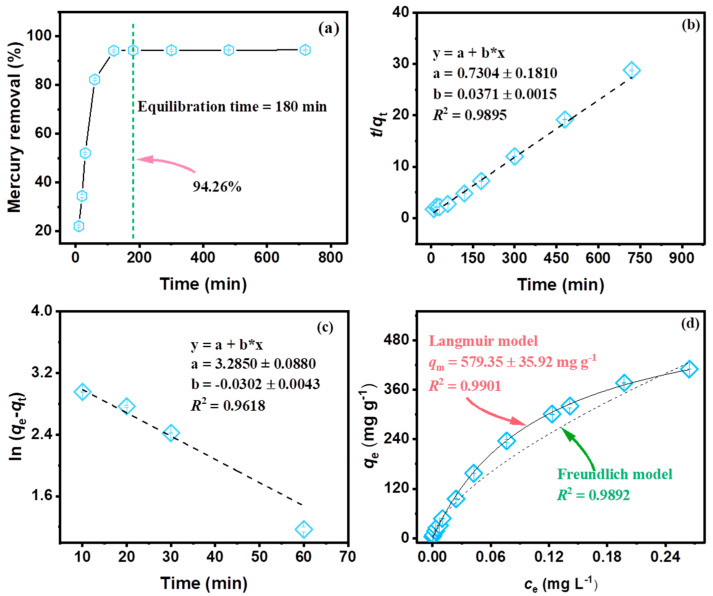
The adsorption of MS for mercury in simulated groundwater: (**a**) adsorption kinetics, (**b**) fitting of pseudo-second order model, (**c**) fitting of pseudo-first order model (MS dosage m = 3.0 mg L^−1^, Hg initial concentration *C*_0_ = 0.08 mg L^−1^, reaction time was 12 h), and (**d**) fitting of Langmuir isotherm and Freundlich isotherm models (MS dosage m = 3.0 mg L^−1^, Hg initial concentration *C*_0_ = 0.01–1.50 mg L^−1^, reaction time was 3 h). (The blue marker are the data points embedded in the error bars).

In order to gain a comprehensive insight into the interfacial adsorption process for mercury, kinetic models including the pseudo-first, the pseudo-second order, and the intraparticle diffusion models were adopted (Figure 4a–c and Figure S2, and Text S5). It can be seen that MS exhibited a shorter equilibrium time (3 h), a higher Hg removal rate (94.26%), and a higher uptake capacity (25.0 ± 0.07 mg g^−1^) (Figure 2a). Furthermore, the distribution of mercury is highly consistent with that of elements Mo and S (Figure 2b,d). The calculated values are presented in Table S1. Compared to the calculated results of the pseudo-first-order and the intraparticle diffusion models, that of the pseudo-second-order model showed a superior fit (*R*^2^ = 0.9895) and a closer adsorption capacity *q*_e_ (27.00 ± 1.54 mg g^−1^). This fully demonstrates that the pseudo-second order model can better describe the sorption kinetics of MS, and the rate-limiting step of the interaction between mercury and MS was mainly governed by chemisorption rather than diffusion [11,24]. The adsorption process can be divided into two stages: surface adsorption of MS and slow pore diffusion; the straight lines do not pass through the origin, indicating that internal diffusion is not the only step controlling the adsorption process (Figure S2). The calculated distribution coefficient of MS for mercury is up to 5.69 × 10^6^ mL g^−1^, which is 56.9 times that of the criterion for excellent adsorbents (1.0 × 10^5^ mL g^−1^) [16]. This indicates that MS is a high-performance adsorbent for mercury.

To better understand the Hg^2+^ sorption process, Hg removal isotherms of MS were obtained with initial concentrations varying from 0.01 to 1.50 mg L^−1^. The uptake of mercury *q*_m_ increased with increasing concentration, which can be attributed to the fact that the driving force provided by the higher initial concentration helps to overcome the resistance of mass transfer from aqueous to solid. However, the mercury removal efficiency decreased (Figure S3). Langmuir isotherm and Freundlich isotherm models were employed to simulate the interfacial sorption of Hg^2+^ (Figure 4d), and the fitted results of the parameters are shown in Table S2. It can be seen that the Langmuir isotherm model (*R*^2^ = 0.9803) can better fit the process, indicating that the interfacial process belongs to monolayer sorption. Furthermore, the calculated maximum adsorption capacities *q*_m_ of MS are as high as 926.10 ± 165.25 mg g^−1^, confirming the high adsorption capacity of MS for Hg even at low concentrations (0.01–1.50 mg L^−1^). The superior performance of MS can be attributed to its nanosheet structure and flower-like shape (Figure 1a), which expose more adsorption sites and provide more access channels for Hg.

### 2.3. Matrix-Dependent Hg Adsorption by MS

**Figure 5:**
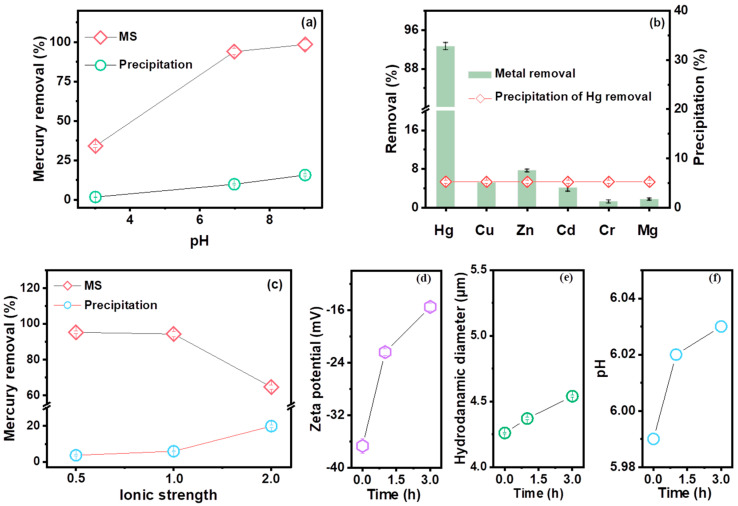
Effects of (**a**) pH, (**b**) coexisting heavy metal ions, (**c**) ionic strength (the value on the horizontal axis represents the dilution time for simulated groundwater) for mercury removal; the changes in (**d**) zeta potential, (**e**) hydrodynamic diameter, and (**f**) pH. Experimental conditions for (**b**): 3.0 mg L^−1^ for MS, the initial concentrations of mercury and each coexisting heavy metal ion are 4.0 × 10^−4^ mmol L^−1^, and for Hg^2+^, the concentration is also 0.08 mg L^−1^.

To investigate the performance of MS for the removal of Hg cations from groundwater matrices, the effects of pH, coexisting metal ions, and ionic strength were studied (Figure 5a–c). Upon increasing the pH from 3.0 to 5.0, the removal efficiency and precipitation showed a consistent increasing trend (Figure 5a), which can be attributed to the gradual strengthening of the ligand exchange (between H^+^ and Hg^2+^) and electrostatic attraction (between MS and Hg^2+^). At pH > 5.0, the formation of Hg(OH)_2_ precipitates gradually increased, facilitating the improvement of the Hg removal efficiency. It is worth noting that under conditions of around pH 8.0, both the predominant soluble mercury (HgClOH (aq., 40.4%) and HgCl_2_ (aq., 7.1%)) and the precipitates Hg(OH)_2_ (45.9%) are favorable factors for improving the removal rate [3,25]. Therefore, the Hg removal efficiency increased from 34.12% to 98.58% as the pH varied from 3.01 to 9.02, accompanied by a precipitation increase from 1.73% to 15.67%. The selectivity of MS toward Hg^2+^ in the presence of coexisting metal ions (Cu^2+^, Zn^2+^, Cd^2+^, Cr^6+^, and Mg^2+^) in simulated groundwater was investigated (Figure 5b). MS showed a higher efficiency for Hg (92.63%), but a lower rate for other metals (1.17–7.58%), mainly due to the high affinity of MS for Hg via Lewis acid–base soft–soft interactions.

**Figure 6:**
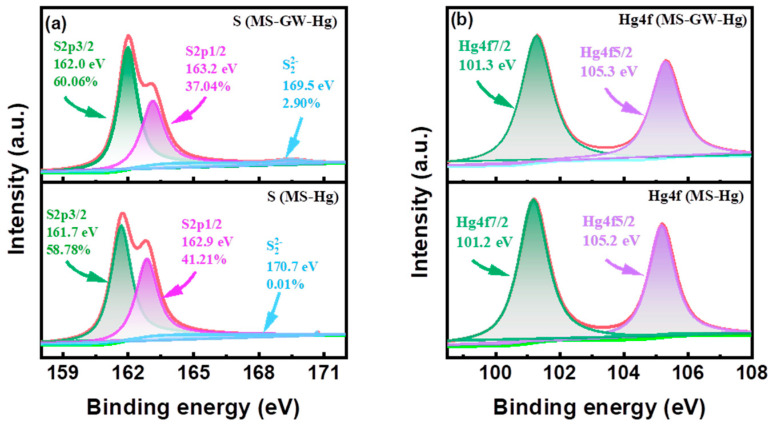
(**a**) High-resolution XPS spectrum of S2p for MS-Hg and MS-GW-Hg; (**b**) high-resolution XPS spectrum of Hg4f for MS-Hg and MS-GW-Hg.

The Hg removal efficiency of MS in groundwater showed a significant decrease (from 95.27% to 64.66%) with increasing ionic strength (Figure 5c), which may be due to cation competition (Ca^2+^ and Na^+^), the shielding of electrically neutral components (e.g., NaCl, Na_2_SO_4_, CaCl_2_, and NaHCO_3_), and the isolation of precipitation (Hg(OH)_2_ and CaCO_3_) [26,27]. Furthermore, the decreasing trend of Hg atomic percentages from 5.02% (MS-Hg) to 0.87% (MS-GW-Hg) can also confirm the inhibition of ionic strength (Figure 2b,d). However, the removal of Hg precipitation increased from 3.63% to 19.95%. On the one hand, high ionic strength facilitates the formation of Hg precipitation (Hg(OH)_2_) and the co-precipitation of Hg^2+^ and Ca^2+^. On the other hand, the correlation between Hg precipitation and HgClOH species at different Cl^−^ concentrations also plays an important role. Specifically, the HgClOH species is the predominate source of soluble Hg in selected groundwater, and the formation of the HgClOH species was promoted by moderate Cl^−^ concentrations (0–600 mg L^−1^), followed by the formation of ^•^HgCl radicals on the MS nanosheets, which can either dimerize into a stable Hg_2_Cl_2_ precipitate or be reduced to Hg^0^ by MS. Moreover, observations were also made on the structure and elemental composition of the material before and after adsorption. Interestingly, the oxidation of MS by dissolved oxygen can be negligible after adsorption both in DI water and in groundwater without mercury, which can be confirmed by the relatively stable fraction of $S_{2}^{2-}$ in MS and MS-GW (Figure 3b); the formation of Hg^0^ by the oxidation of MS, driven by the ^•^HgCl radicals originating from HgClOH, also play a minimal role, which can be confirmed by the small increase in $S_{2}^{2-}$ in the high-resolution XPS spectrum of MS-Hg and MS-GW-Hg (Figure 6a). The latter may be attributed to the low concentration of Hg in groundwater. Consequently, the Hg removal of precipitation increased with increasing ionic strength. Compared to the morphology of MS-Hg, the presence of more particles and Cl atoms on MS-GW-Hg may also confirm the formation of Hg_2_Cl_2_ precipitation (Figure 1 and Figure 2b,d).

Due to the negative charge on the MS surface, the abundant active adsorption sites of S, and the unique structure of MS, the high selective adsorption of MS for positive mercury species (e.g., Hg^2+^, HgCl^+^, and HgOH^+^) or uncharged mercury species (e.g., Hg(OH)_2_, HgClOH, and HgCl_2_) in simulated groundwater leads to an increase in zeta potential and hydraulic diameter (Figure 5d–f and Figure S4).

### 2.4. Column Hg Removal of MS

**Figure 7:**
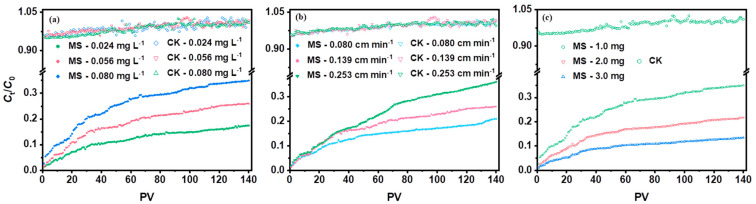
Effects of (**a**) concentration of Hg in groundwater (initial concentration *C*_0_ = 0.024–0.080 mg L^−1^, pore velocity *ν*_p_ = 0.139 cm min^−1^, 25 ± 1 °C, dosage m =1.0 mg), (**b**) influent pore velocity (pore velocity *ν*_p_ = 0.080–0.253 cm min^−1^, initial *C*_0_ = 0.056 mg L^−1^, 25 ± 1 °C, dosage m = 1.0 mg), and (**c**) adsorbent dosage (dosage m = 1.0–3.0 mg; initial concentration *C*_0_ = 0.080 mg L^−1^, 25 ± 1 °C, pore velocity *ν*_p_ = 0.139 cm min^−1^) on the breakthrough curve of Hg adsorption on MS in the fixed-bed column.

In general, the values of *C*_t_/*C*_0_ increase with increasing pore volumes (PVs) in the column (Figure 7), implying that the available adsorption active sites on MS gradually decrease as the flowing groundwater continues to infiltrate. After 140 PVs (the total volume of injected groundwater is 1113 mL), the *C*_t_/*C*_0_ values for MS reached 13.43–36.14%. Furthermore, the variables such as the initial concentration of injected groundwater, influent pore velocity, and adsorbent dosage can significantly affect the Hg interception of MS. Control tests showed that the effects of quartz sand and column materials on Hg removal can be ignored (<5%).

There is a significant correlation between the breakthrough time (*t*_b_) and the operating parameters (*C*_0_, *ν*_p_, and *m*) for the column test of MS. As the influent concentration *C*_0_ increased from 0.024 to 0.080 mg L^−1^, the *t*_b_ decreased sharply from 10.90 years (78,696 PVs) to 1.00 years (7220 PVs), and the adsorption capacity increased from 22.0 to 57.5 mg g^−1^ (for 140 PVs) (Figure 7a and Table S3). Due to the higher mass transfer driving force and the higher diffusion coefficient driven by the high Hg concentration, more mercury was transported from the aqueous to the MS surface [2,28]. However, a correspondingly shorter breakthrough time was achieved. Similarly, the *t*_b_ dropped from 15.87 years (114,578 PVs) to 0.16 years (1155 PVs) with increasing pore velocities *ν*_p_ from 0.080 to 0.253 cm min^−1^ (increasing adsorption capacity from 39.6 to 48.9 mg g^−1^) (Figure 7b and Table S3). This can be attributed to the fact that a higher velocity can weaken the thickness of the external mass transfer film by increasing the mass transfer coefficient, but lower velocity provides a longer residence time for the Hg transfer to the internal sites on MS, resulting in more occupiable active sites on the sorbent. In terms of dosage, the *t*_b_ increased sharply (from 1.00 years to 15.44 years) as the dosage varied from 1.0 to 3.0 mg (Figure 7c and Table S3). Conversely, the increase in dosage inhibited the improvement of the adsorption capacity due to the relative surplus of available adsorption sites on SM.

### 2.5. Mathematical Model of Breakthrough Curves

The fitted results are listed in Table S3. The Adams–Bohart model (*R*^2^ = 0.9052–0.9416) can better describe the dynamic process of Hg interception in the initial stage (≤40 PVs). For 140 PVs, the Yan model (*R*^2^ = 0.0.9765–0.9941) can more accurately depict the process than the other three models. Based on the results fitted according to the Yan model, the maximum adsorption capacity *q*_Y_ can reach up to 1097.3 mg g^−1^, the *t*_b_ (95% breakthrough time) can be up to 15.87 years, and the *p*_τ_ (pore volume of 50% breakthrough) ranges from 282.15 to 2478.0 PVs, indicating that MS has an excellent adsorption capacity for mercury in groundwater.

### 2.6. Plausible Adsorption Mechanism

Compared to MS-Hg (3 elements), the elements in MS-GW and MS-GW-Hg have increased to 7 and 8, respectively, due to the groundwater matrix (Figure 2b,d), and MS still has a high removal efficiency for mercury in low Hg-contaminated groundwater. In addition, the Hg uptake of MS (926.10 ± 165.25 mg g^−1^) is higher than most similar materials recently reported [11,13,20,21,29,30,31,32,33] (Table S4). This confirms that MS has high selectivity for Hg. XPS and SEM-mapping were employed to characterize the chemical composition of MS before and after Hg uptake. New peaks located at 101.2–101.3 eV and 105.2–105.3 eV in the Hg-laden MS are attributed to Hg4f7/2 and Hg4f5/2 respectively, indicating the presence of Hg in MS (Figure 3a and Figure 6b). In the order of MS, MS-GW, MS-Hg, and MS-GW-Hg, the contribution of S2p1/2 increased significantly from 28.37% to 42.21%, but that of S2p3/2 decreased correspondingly from 68.96% to 58.78% (Figure 2). The strong Hg-S complexation between Hg species (HgClOH, Hg(OH)_2_, and HgCl_2_) in groundwater and the S-active adsorption site in MS plays a major role [3,34,35]. Considering the surface electronegativity, the electrostatic attraction to cations (Hg^2+^, HgOH^+^, Ca^2+^, Na^+^, HgCl^+^, and CaHCO_3_^+^) by MS is also an important reason. From MS to MS-GW-Hg, the atomic ratio of S/Mo increased from 2.04 to 2.25, indicating that partial Mo was exchanged by target ions to form stable complexes (Figure 2). Compared to other cations, S has a stronger binding affinity for Hg, so the subsequent ion exchange is also inevitable. In addition, the gradual precipitation of the Hg species (Hg(OH)_2_ and HgSO_4_) and co-precipitation with calcium species (CaCO_3_ and CaSO_4_) may also occur.

## 3. Materials and Methods

All chemicals were at least analytical grade and were used without further purification. Detailed information on chemicals and characterizations is provided in the Supplementary Material (Text S1).

Flower-like MS was prepared by a simple one-step hydrothermal–solvothermal method according to our previous work [11]. Firstly, 0.56 mmol (NH_4_)_6_Mo_7_O_24_•4H_2_O and 20 mmol CH_4_N_2_S were dissolved in 50 mL DI water, and the resulting mixture was dispersed by ultrasonic treatment for 20 min to form a homogeneous solution. The solution obtained was then transferred to a Teflon-lined stainless steel autoclave and heated at 190 °C for 12 h. Finally, the black solid was centrifuged, washed six times with DI water, and dried in a vacuum (60 °C, 12 h), and the MS was obtained.

Batch tests of Hg^2+^ adsorption in groundwater were performed in 50 mL polytetrafluoroethylene (PTFE) vials. Specifically, an appropriate amount of MS suspension was added to the simulated groundwater. The solid–liquid mixture was then vortexed at 200 rpm, and the reactive time was 3 h (25 ± 1 °C), followed by filtration (0.22 μm, PTFE). The resulting filtrate was used for further analysis. For sorption kinetics, the initial Hg^2+^ concentration was 0.08 mg L^−1^, the MS dosage was 3.0 mg L^−1^, and the pH was adjusted to about 6.0. For the sorption isotherms, the concentrations of Hg^2+^ varied from 0.01 to 1.50 mg L^−1^. In addition, the effect of several factors such as pH (3.0–9.0), ionic strength (the dilution was 0.5–2.0), and co-existing ions (Cu, Zn, Cd, Cr, and Mg) were also investigated. The pH of simulated groundwater was determined to be 8.08 ± 0.2. The removal efficiency *R* (%) and the adsorption capacity *q*_e_ (mg g^−1^) are given in Text S2. Control tests without MS (named CK) showed that the loss of Hg during the process was <5%. All experiments were carried out in triplicate.

The column tests were carried out in a specific experimental set-up including injection pump (PHD/ULTRA, Harvard Apparatus, Houston, MA, USA), syringe (diameter = 35.57 mm, volume = 100 mL), column (006EZ-10-25-FF, Omnifit, Amersham, Buckinghamshire, UK), automatic sample collector (BSZ–160, Jiapeng, Shanghai), and connecting tube (PTFE). There are four layers inside the 22 cm high column (ordered from top to bottom): quartz sands (porosity = 0.33, h = 10.0 cm); 1 mg MS (mixed with 0.5 g sands, h = 0.5 cm); quartz sands (porosity = 0.33, h = 11.2 cm); glass wool (0.2 g, h = 0.3 cm). The pore volume (PV) (the total volume within the column excluding that of MS sorbents, sands, and glass wool) of the column was determined to be 7.95 mL. Groundwater was injected into the column with a quantitative flow, and the filtrate was collected by a collector. A glass wool/sand control column was operated. Column tests were performed under different operating conditions, such as influent Hg^2+^ concentration (*C*_0_ = 0.02–0.08 mg L^−1^), groundwater pore velocity (*ν*_p_ = 0.080–0.253 cm min^−1^), and MS dosage (m = 1.0–3.0 mg). To quantify the interception efficiency of MS for Hg, the uptake amount of Hg in the fixed-bed column (*q*_t_, mg g^−1^) was employed, and the calculation of *q*_t_ is detailed in Text S3.

Mathematical models were used to describe the influence of operational variables, such as Hg^2+^ concentration, pore velocity, and MS dosage, on column interception performance. Four models were utilized to describe the breakthrough curves, including the Yan model, the Adams–Bohart model (*C*_t_/*C*_0_ < 0.5), the Yoon–Nelson model, and the Thomas model [2,3,36,37,38,39,40], as follows:

Yan model

(1) $$\frac{C_{t}}{C_{0}}=1-\frac{1}{1+\left(\frac{C_{0}V_{0}n}{1000q_{Y}m}\right)}$$

Adams–Bohart model

(2) $$\frac{C_{t}}{C_{0}}=\exp\left(\frac{K_{\mathrm{AB}}C_{0}V_{0}}{Q}n-\frac{K_{\mathrm{AB}}N_{0}H}{\nu_{p}}\right)$$

Yoon–Nelson model

(3) $$\frac{C_{t}}{C_{0}}=\frac{1}{1+\exp\left(\frac{K_{\mathrm{YN}}V_{0}p_{\tau }-K_{\mathrm{YN}}V_{0}n}{Q}\right)}$$

Thomas model

(4) $$\frac{C_{t}}{C_{0}}=\frac{1}{1+\exp\left(\frac{1000K_{\mathrm{Th}}q_{\mathrm{Th}}m-V_{0}K_{\mathrm{Th}}C_{0}n}{1000Q}\right)}$$

Detailed information on *C*_t_/*C*_0_, *K*_AB_, *C*_0_, *V*_0_, *H*, *Q*, *n*, *ν*_p_, *K*_Th_, *q*_Th_, *m*, *K*_YN_, *p*_τ_, *K*_Y_, and *q*_Y_ is provided in Text S4. The coefficient of determination (*R*^2^) was accepted to assess the fit of the models representing the dynamic Hg interception of MS in PRBs.

## 4. Conclusions

In summary, flower-like MS with nanosheets exhibited high selectivity and superior adsorption ability for low-concentration mercury in a high-salinity groundwater matrix. Our study demonstrated that MS can efficiently remove Hg from groundwater without significant interference from factors such as pH variations, coexisting metal ions (Cu^2+^, Zn^2+^, Cd^2+^, Cr^6+^, and Mg^2+^), and high ionic strength in the groundwater matrix. In addition, some constituents in selected groundwater, including Ca^2+^, Cl^−^, SO_4_^2−^, and HCO_3_^−^ in the matrix, are beneficial for mercury removal. Hg species (HgClOH, Hg(OH)_2_, and HgCl_2_) in the groundwater matrix can be effectively intercepted by MS due to surface complexation, electrostatic attraction, ion exchange, and precipitation. The kinetic process has a shorter equilibrium time (3 h), higher removal rate (94.26%), and excellent distribution coefficient (5.69 × 10^6^ mL g^−1^). The fitted result isotherm model shows that the calculated maximum adsorption capacity *(q*_m_) can be up to 926.10 ± 165.25 mg g^−1^. The Yan model can accurately depict the process, and the maximum adsorption capacity *q*_Y_ can reach up to 1097.3 mg g^−1^, and the *t*_b_ (95% breakthrough time) can be up to 15.87 years. These practical advantages, including superior selectivity, high uptake, excellent anti-interference with complex substrates, and longer penetration time, demonstrate that MS has great potential for the practical PRB remediation of Hg-contaminated groundwater.

## Data Availability

Data is contained within the article or Supplementary Materials.

## Supplementary Materials

The following supporting information can be downloaded at: https://www.mdpi.com/article/10.3390/molecules29215132/s1. Figure S1: XRD patterns of MS, MS-Hg, MS-GW, and MS-GW-Hg. Figure S2: Fitting of Weber-Morris model. Figure S3: Mercury adsorption removal efficiencies (R) at multiple initial Hg^2+^ concentrations after mercury uptake from simulated groundwater (m = 3.0 mg L^−1^ adsorbent, C_0_ = 0.01~1.50 mg L^−1^, reaction time was 180 min). Figure S4: Zeta potential at different pH values. Table S1: Pseudo-first-order, pseudo-second-order kinetic and Weber-Morris models used for simulating Hg sorption kinetic data and the resulting fitting parameters. Table S2: Fitting parameters of Langmuir and Freundlich isotherm models for Hg sorption. Table S3: Parameters fitted by Adams-Bohart, Thomas, Yoon-Nelson, and Yan models for Hg adsorption by MS in the fixed-bed column. Table S4: Comparison of the uptake of mercury by different adsorbents.
