# Supplementary material for: Practical Remediation of Hg-Contaminated Groundwater by MoS2: Batch and Column Tests
Source: Molecules. 2024 Oct 30;29(21):5132. doi: 10.3390/molecules29215132 (PMC11547822; doi:10.3390/molecules29215132)
Supplement: Supplementary file 1 [file molecules-29-05132-s001.zip › molecules-3263924-supplementary.pdf]

Practical remediation of Hg-contaminated groundwater by MoS_2: Batch and column tests

Haifeng Wang 1, Shuai Wei 2, Shuai Huang 1, Wei Liu 1 and Zongwu Wang 1,*

1 Kaifeng Key Laboratory of Food Composition and Quality Assessment, School of Environmental

Engineering, Yellow River Conservancy Technical Institute, Kaifeng 475004, China

2 Department of Science and Technology Evaluation Service, Henan Provincial Science Research Platform

Service Center, Zhengzhou 450008, China

* Correspondence: kf0986@163.com

Total number of pages of Supporting Information: **10**

Number of Texts in Supporting Information: **6**

Number of Tables in Supporting Information: **4**

Number of Figures in Supporting Information: **4**

Text S1

Materials: Ammonium molybdate tetrahydrate ($(NH_4)_6Mo_7O_{24}$•$4H_2O$), thiourea (CH_4N_2S) were purchased from Aladdin Reagents. Mercury nitrate monohydrate ($Hg(NO_3)_2 \cdot H_2O$) was obtained from Sinopharm Chemical Reagent Co.Ltd (Shanghai, China). Reference material (RM) of Hg (1000 mg L^{-1} in 5% HNO_3) was obtained from Tan-Mo Technology Co., Ltd (Beijing, China). NaCl, Na_2SO_4, $CaCl_2$, $NaHCO_3$ and Quartz sand (20~40 mush) were procured from Damao chemical reagent factory (Tianjin, China). Potassium hydroxide (KOH) (AR), potassium borohy-dride (KBH_4) (AR), glucose (98%), and humic acid (98%) were obtained from Macklin Reagent Co.Ltd (Shanghai, China). Hydrochloric acid (HCl) and sodium hydroxide (NaOH) (AR) were obtained from Tianjin Chemical Reagent Technology (Tianjin, China). All solutions were prepared with DI water (18 MΩ·cm). The quartz sands were pretreated following the reported approach.

The simulated groundwater in this study contained Hg^{2+} (0.08 mg L^{-1}), Na^{+} (230.0 mg L^{-1}), Ca^{2+} (32.0 mg L^{-1}), Cl^{-} (234.3 mg L^{-1}), $SO4^{2-}$ (96.0 mg L^{-1}), and HCO_3^{-} (183.0 mg L^{-1}). The simulated groundwater was prepared and filtered by PTFE membrane (0.22 μm) before use. The initial pH was adjusted with HCl and NaOH.

Characterizations: Structure and morphology were observed by scanning electron microscopy (SEM) (Sigma300, Carl Zeiss). The surface atomic ratio and the binding energy of Mo3d, S2p, Na1s, Ca2p, C1s, O1s, Cl2p and Hg4f were analyzed by X-ray photoelectron spectrometer (XPS) (K-alhpa, Thermo Fisher) with Al-Kα radiation (pass energy 150.0 eV). X-ray diffraction (XRD) pattern was collected on Bruker D8-AXS. The Zeta potential and particle size distribution were determined using a Zeta potential and nanoparticle size analyzer (Nanotrac wave II, Microtrac). The pH was measured with a pH meter (FE28 stander, Mettler Toledo). The concentration of Hg was determined according to the environmental protection standards of the People's Republic of China (HJ 694-2014) by atomic fluorescence spectrometer (AFS) (BAF-4000, Baode) with a detection limit of 0.04 μg L^{-1}. The concentrations of other metal ions were determined following the trade standard of HJ 776-2015 (China) by inductively coupled plasma optical emission spectrometer (ICP-OES) (PlasmaQuantPQ 9000, Analytik Jena). Mercury and calcium ion speciation in simulated groundwater was conducted using Visual MINTEQ 3.1.3.

Text S2

The adsorption capacity q_e (mg g^{-1}) and removal efficiency of MS was calculated according the following equations:

$$q_{\mathrm{e}} = \frac{(C_0 - C_e)\times V}{m} \quad \text{(S1)}$$

$$R\% = \frac{C_0 - C_e}{C_0}\times 100\% \quad \text{(S2)}$$

where V (L) is the volume of the groundwater, C_0 and C_e (mg L^{-1}) are the initial and equilibrium concentrations of the metal ions, m (g) is the mass of MS used.

Text S3

The t_b is the time of 95% breakthrough (C_t/C_0 = 0.95). The total amount of mercury sorbed in the column (q_t, mg g^{-1}) can be calculated according to the following equation (S3) at a given influent mercury concentration (C_0):

$$q_{\mathrm{t}} = \int_0^{V_{\mathrm{t}}} \frac{C_0 - C_{\mathrm{t}}}{m} dV \quad \text{(S3)}$$

where C_t (mg g^{-1}) is the effluent Hg^{2+} concentration, V (L) is the volume of mercury contaminated groundwater, V_t (L) is the total volume of groundwater when the effluent mercury concentration reaches C_t, and m (g) is the mass of sorbent in column.

Text S4

(1) Yan model

$$\frac{C_{\mathrm{t}}}{C_0} = 1 - \frac{1}{1 + \left(\frac{C_0 V_0 n}{1000 q_{\mathrm{Y}} m}\right)} \quad \text{(S4)}$$

where q_Y (mg g^{-1}) is the maximum adsorption capacity; K_Y is the constant of Yan model; other parameters have the same meaning as above. The values of K_Y and q_Y can be evaluated from a plot of C_t/C_0 against n using non-linear regression analysis.

(2) Adams-Bohart model

$$\frac{C_{\mathrm{t}}}{C_0} = \exp\left(\frac{K_{\mathrm{AB}} C_0 V_0}{Q} n - \frac{K_{\mathrm{AB}} N_0 H}{v_{\mathrm{p}}}\right) \ (C_t/C_0<0.5) \quad \text{(S5)}$$

where K_{AB} (L min^{-1} mg^{-1}) is the Adams-Bohart model rate constant; N_0 (mg L^{-1}) is the saturated adsorption capacity per column volume; H (cm) is the bed depth of the column; v_p (cm min^{-1}) is the pore velocity; n is the number of pore volumes; V_0 (mL) is one pore volume of the packed column (7.95 mL in this study); and Q (mL min^{-1}) is the volumetric flow rate; C_0 (mg L^{-1}) and C_t (mg L^{-1}) are the mercury concentrations in the influent and at time t (min), respectively. t =

$n \times V_0/Q$. The values of K_{AB} and N_0 can be evaluated from a plot of C_t/C_0 against n by non-linear regression analysis.

(3) Yoon-Nelson model

$$\frac{C_t}{C_0} = \frac{1}{1+\exp\left(\frac{K_{YN}V_0 p_\tau - K_{YN}V_0 n}{Q}\right)} \tag{S6}$$

where K_{YN} (min^{-1}) is the Yoon-Nelson kinetic constant; and p_τ represents the number of pore volumes at time τ (the contact time required 50% adsorbate breakthrough, C_t/C_0 = 0.5); other parameters have the same meaning as above. The values of K_{YN} and p_τ can be evaluated from a plot of C_t/C_0 *vs*. n using non-linear regression analysis as the values of C_t/C_0 are within 0.05–0.95.

(4) Thomas model

$$\frac{C_t}{C_0} = \frac{1}{1+\exp\left(\frac{1000K_{Th}q_{Th}m - V_0 K_{Th} C_0 n}{1000Q}\right)} \tag{S7}$$

where K_{Th} (mL min^{-1} mg^{-1}) is the Thomas rate constant; q_{Th} (mg g^{-1}) is the equilibrium sorption capacity; m (g) is the amount of sorbent in the column; other parameters have the same meaning as above. The values of K_{Th} and q_{Th} can be determined from a plot of C_t/C_0 *vs*. n at given experimental conditions using non-linear regression analysis.

Text S5

The pseudo-first-order, the pseudo-second-order and the Weber-Morris models are described as Eqs. (S8)-(S10), respectively:

$$\ln(q_e - q_t) = \ln q_e - K_1 t \tag{S8}$$

$$\frac{t}{q_t} = \frac{t}{q_e} + \frac{1}{K_2 q_e^2} \tag{S9}$$

$$q_t = K_{id} t^{1/2} + C \tag{S10}$$

where q_t (mg g^{-1}) is uptake of Hg^{2+} by adsorbents at time t (min), $q = (C_0 - C_e) \times V/m$, C_0 and C_e are the initial and equilibrium concentrations of Hg^{2+}. V is the volume of the solution (L), and m is the mass of adsorbent (mg), q_e is the uptake of Hg^{2+} at equilibrium, K_1 is the rate constant of the pseudo-first-order sorption, K_2 is the pseudo-second-order sorption rate constant, and K_{id} is the internal diffusion coefficient. The governing equations of the two models are integrated by applying the boundary conditions q = 0 (t = 0). Pseudo-first-order was based on the theory of

membrane diffusion, and the adsorption rate is related to the difference between the equilibrium adsorption uptake and the adsorption uptake. Pseudo-second-order was established on the adsorption rate limiting step, involving electron sharing or electron transfer between the adsorbate and the adsorbent, representing chemical adsorption. All kinetic parameters were calculated through nonlinear regression using OriginLab 2021 software program.

Text S6

The Langmuir isotherm model and Freundlich isotherm model (Eq. (S11) and (S12)) are respectively as the following:

$$q_{\mathrm{e}} = \frac{q_{\mathrm{m}} b_{\mathrm{L}} C_{\mathrm{e}}}{1 + b_{\mathrm{L}} C_{\mathrm{e}}} \tag{S11}$$

$$q_{\mathrm{e}} = K_{\mathrm{f}} C_{\mathrm{e}}^{n} \tag{S12}$$

Where q_{e} is the uptake of Hg Hg^{2+} at equilibrium (mg g^{-1}), C_{e} is the equilibrium aqueous mercury concentration (mg L^{-1}). Langmuir isotherm: q_{m} is the maximum sorption capacity, b_{L} is the Langmuir constant related to the free energy of adsorption. Freundlich isotherm: K_{f} is a constant related to the adsorption capacity of the sorbent, n is the adsorption intensity or the heterogeneity of the sorbent. Langmuir isotherm model depicts monolayer sorption, and Freundlich isotherm model describes nonideal sorption on heterogeneous surfaces. All isotherm parameters were calculated through nonlinear regression using OriginLab 2021 software program.

Table S1 Pseudo-first-order, pseudo-second-order kinetic and Weber-Morris models used for simulating Hg sorption kinetic data and the resulting fitting parameters.

Kinetic model	Parameters	Values
Pseudo-first-order $\ln(q_e - q_t) = \ln q_e - K_1 t$	K_1 (min^{-1})	$(3.02\pm0.43)\times10^{-2}$
	q_e (mg g^{-1})	26.81±3.33
	R^2	0.9618
Pseudo-second-order $\frac{t}{q_t} = \frac{t}{q_e} + \frac{1}{K_2 q_e^2}$	K_2 (g (mg·min)$^{-1}$)	$(2.01\pm0.07)\times10^{-3}$
	q_e (mg g^{-1})	27.00±1.54
	R^2	0.9895
	K_d (mL g^{-1})	5.69×10^{6}
Weber-Morris model $q_t = K_{id} t^{1/2} + C$	K_{id}	0.5700±0.2091
	R^2	0.5149

Note: K_d is the distribution coefficient at adsorption equilibrium, which can be calculated by the formula $K_d = \frac{(C_0 - C_e)\times V}{C_e \times m}$.

Table S2 Fitting parameters of Langmuir and Freundlich isotherm models for Hg sorption.

Adsorption isotherm	Parameters	SP
Langmuir model $q_e = \frac{q_m b_L C_e}{1 + b_L C_e}$	q_m (mg g^{-1})	926.10 ± 165.25
	b_L (L mg^{-1})	12.60 ± 3.04
	R^2	0.9803
Freundlich model $q_e = K_f C_e^n$	K_f (mg g^{-1})/(mg L^{-1})^n	$(3.30 \pm 0.449)\times10^{2}$
	n	0.76 ± 0.03
	R^2	0.9780

Note: All isotherm parameters were calculated through by non-linear regression using Origin 2021 software program.

Table S3 Parameters fitted by Adams-Bohart, Thomas, Yoon-Nelson, and Yan models for Hg adsorption by MS in the fixed-bed column.

Variables	Adams-Bohart model			Thomas model			Yoon-Nelson model			Yan model					Uptake of Hg
	K_{AB}(×10^{-3}) (L min^{-1} mg^{-1})	N_0 (mg L^{-1})	R^2	K_{Th} (mL min^{-1} mg^{-1})	q_{Th} (mg g^{-1})	R^2	K_{YN}(×10^{-4}) (min^{-1})	p_τ	R^2	K_Y	q_Y (mg g^{-1})	R^2	t_b year	p_τ	q_t mg g^{-1}
C_0 (mg L^{-1})[a]															
0.080	5.32	3.10	0.9183	1.62	117.1	0.8161	1.29	185.3	0.8161	0.5652	532.7	0.9848	1.00	382.1	22.0
0.056	5.79	3.37	0.9105	2.27	104.3	0.8287	1.26	235.5	0.8287	0.5631	367.5	0.9864	2.13	829.9	45.9
0.024	5.80	3.77	0.9076	4.79	59.5	0.8288	1.15	311.7	0.8288	0.5204	241.5	0.9861	10.90	2744.7	57.5
v_p (cm min^{-1})[b0.]															
0.253	15.17	2.27	0.9468	5.51	75.90	0.8965	1.69	171.4	0.8965	0.7855	124.9	0.9941	0.16	282.15	48.9
0.139	5.79	3.37	0.9105	2.27	104.3	0.8287	1.26	235.5	0.8287	0.5631	367.5	0.9864	2.13	829.9	45.9
0.080	4.30	8.44	0.9052	1.13	132.7	0.8288	1.09	299.6	0.8288	0.4809	1097.3	0.9825	15.87	2478.0	39.6
m (mg)[c]															
1.0	5.32	3.10	0.9183	1.62	117.1	0.8161	1.29	185.3	0.8161	0.5652	532.7	0.9848	1.00	382.1	57.5
2.0	5.80	3.45	0.9357	1.40	88.8	0.7974	1.11	281.0	0.7974	0.4960	452.7	0.9874	9.16	1749.0	34.6
3.0	5.78	3.98	0.9416	1.28	79.0	0.7946	1.02	375.0	0.7946	0.4603	391.4	0.9765	15.44	1857.9	25.5

Note: (1) The results of Thomas, Yoon-Nelson, and Yan models were fitted using the data from 140 PVs, and that of Adams-Bohart model was fitted using the data from 40 PVs. p_τ is the number of pore volumes at 50% adsorbate breakthrough (C_t/C_0 = 0.5); q_t (mg g^{-1}) is the Hg adsorption capacity of adsorbent MS; R^2 is the coefficients of determination. Detailed information is shown in Text S4.

(2) Experimental conditions:

[a]: v_p = 0.139 cm min^{-1}, m = 1.0 mg, 25 ± 1 °C;

[b]: C_0 = 0.056 mg L^{-1}, m = 1.0 mg, 25 ± 1 °C;

[c]: C_0 = 0.080 mg L^{-1}, v_p = 0.139 cm min^{-1}, 25 ± 1 °C

Table S4 Comparison of the uptake of mercury by different adsorbents.

**Adsorbents**	q_{max}	**Ref.**
MoS_2/Fe_3O_4	425.5	[13]
CS-PAM-MCM	263.9	[29]
MAF-SCMNPs	355	[30]
Fe_3O_4@MOF	348.43	[31]
rGO-PDTC/Fe_3O_4	181.82	[32]
rGO-p($C_3N_3S_3$)/Fe_3O_4	400.0	[33]
MoS_2	428.9	[11]
P-PVDF/MoS_2	578	[20]
double-network MoS_2-based beads (DMBs)	253.6	[21]
MoS_2	926.10 ± 165.25	Present work

Figure S1

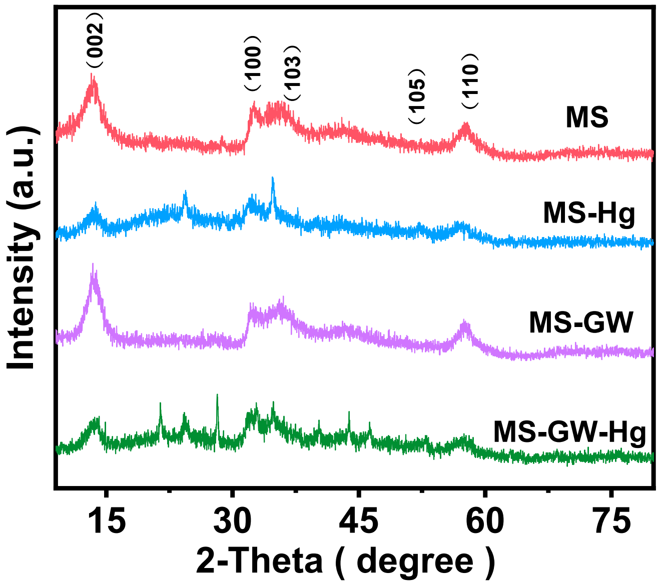

Figure S1. XRD patterns of MS, MS-Hg, MS-GW, and MS-GW-Hg.

Fig. S2

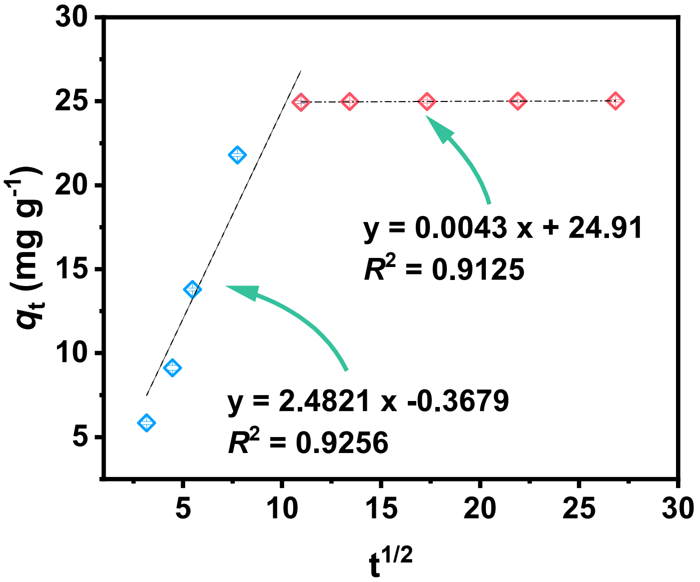

Figure S2. Fitting of Weber-Morris model.

Fig. S3

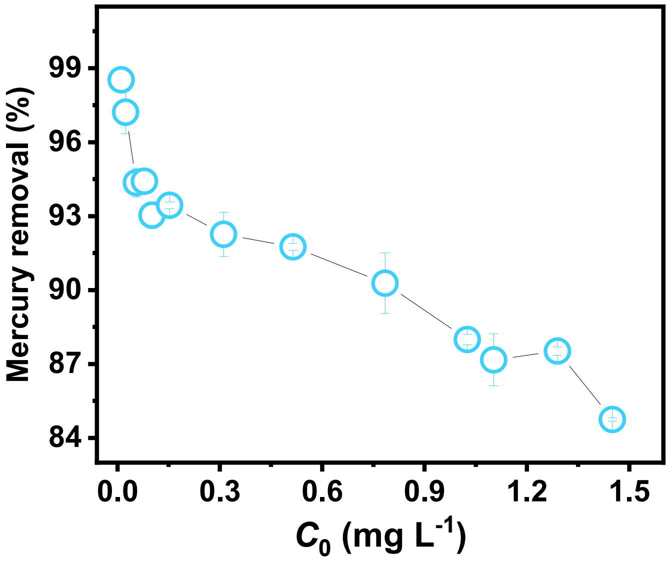

Figure S3. Mercury adsorption removal efficiencies (*R*) at multiple initial Hg^{2+} concentrations after mercury uptake from simulated groundwater (m = 3.0 mg L^{-1} adsorbent, C_0 = 0.01 ~ 1.50 mg L^{-1}, reaction time was 180 min).

Fig. S4

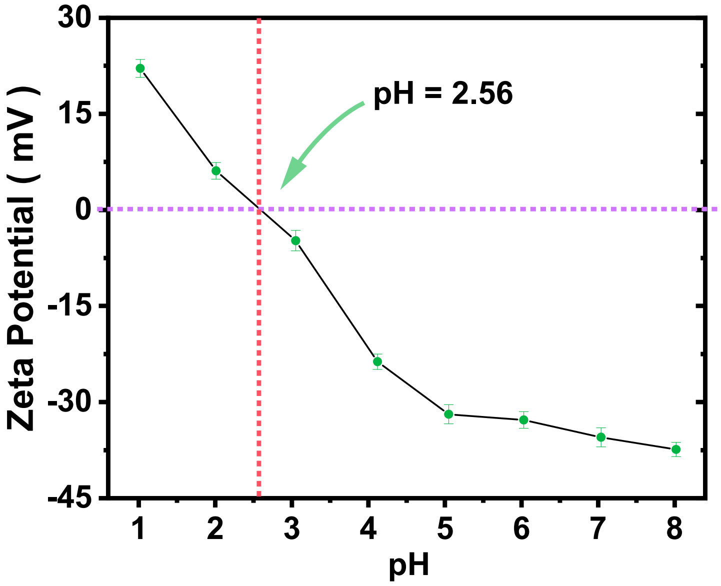

Figure S4. Zeta potential at different pH values.
